# Sonographers’ experiences of being a caring professional within private practice in the province of Gauteng

**DOI:** 10.4102/hsag.v25i0.1409

**Published:** 2020-12-07

**Authors:** Leah van der Westhuizen, Kathleen Naidoo, Yasmin Casmod, Sibusiso Mdlethse

**Affiliations:** 1 Medical Imaging and Radiation Sciences, Faculty of Health Sciences, University of Johannesburg, Johannesburg, South Africa; 2 Medical Imaging and Therapeutic Sciences, Health and Wellness Sciences, Cape Peninsula University of Technology, Cape Town, South Africa; 3 Department of Anatomy and Medical Imaging, University of Auckland, Auckland, New Zealand

**Keywords:** caring, healthcare professional, phenomenology, qualitative, sonographer, sonography

## Abstract

**Background:**

Medical imaging has been driven by technological advancements. However, the concept of caring has now become a significant element in the healthcare profession. Within a South African context, there are principles that emphasise the importance of people and service delivery: the Batho Pele Principles and Ubuntu. Now more than ever, there is a greater need for a patient-centred caring environment. Therefore, there is an expectation for sonographers to be adaptive to this new environment.

**Aim:**

The purpose of this study was to explore and describe the sonographers’ experiences of being caring professionals.

**Setting:**

Sonographers who work within private practices in Gauteng were part of this study.

**Methods:**

A qualitative, exploratory, descriptive, contextual, phenomenological research design was used. Focus group interviews were conducted with qualified sonographers registered with the HPCSA. Thematic analysis was used to code data into themes and categories.

**Results:**

Four themes emerged: the effects of a caring relationship between a sonographer and a patient; circumstances limiting a sonographer in being a caring professional; sonographers’ approach to caring; educational readiness of sonographers to be caring professionals.

**Conclusion:**

The participants in this study explained and shared their understanding of being caring professionals. They shared many stories regarding trusting relationships with patients. However, as a caring professional, many challenges were encountered, both physically and emotionally. Recommendations such as reflective journals and the practices of Jean Watson’s theory of transpersonal caring are cited to focus on the practice and education that may improve caring among sonographers.

## Introduction

Sonography is a multifaceted area of practice which consists of a wide range of applications (Gibbs 2013). It is a subdivision of special imaging modalities and is performed by a sonographer (Dupree 2017; Ehrlich & Daly 2009). A sonographer is a healthcare professional who has learned the skill of producing images of diagnostic quality (Sanders & Winter 2007). Sonographers comment on the findings of the sonography examination and suggest additional clinical examinations or follow-up procedures (Sanders & Winter 2007). Sonographers are therefore required to have constant, individual and prolonged interaction with patients. During this time of interaction, the sonographer is expected to be a caring professional.

Caring is the moral ideal of a healthcare professional (Baldursdottir & Jonsdottir 2002). The continuous interaction with patients can be an immensely rewarding experience, and it is what that keeps many in this field (Mathieu 2007). Nadelson et al. (2016) describe a caring professional as someone who connects with people in a dynamic and empathetic manner. It is being aware of the patient, reflecting on the responses of the patient and understanding the patient’s perceptions (Quirk et al. 2008). There are various opinions on caring, and although the expressions vary amongst cultures, there is a universal agreement that caring is based on communication and relationship building among patients and the healthcare professional (Nadelson et al. 2016). Paulson (2004) articulates that patient care and caring are two different concepts. Patient care is associated with physiological and medical care. Conversely, caring for patients is described as a humanistic manner of sincerely caring and interacting with patients (Quirk et al. 2008). Patient-centred care focuses on understanding and treating the patient in a holistic fashion as a unique human being (Fix et al. 2018). It therefore emphasises the importance of respecting and meeting the standards and needs of patients.

Patient satisfaction is gradually becoming important as a marker of healthcare quality (Howard et al. 2014). Patients often describe their healthcare experience based on their interaction with a healthcare professional (Bolderston 2016). The strong climate and professional acts of caring within the healthcare organisation are no longer a convenience, but rather an essential aspect of the success of a healthcare organisation (Canfield et al. 2016; Quirk et al. 2008).

Within a South African context, healthcare has two distinctive principles that focus on the patient, that is, Batho Pele and Ubuntu. Batho Pele is a Sesotho phrase that translates into ‘people first’ and aims for excellence in service delivery (Department of Public Service and Administration, Republic of South Africa 2014; Pietersen 2014). De la Porte (2016) describes the African worldview as a holistic one characterised by a strong community bond, a sharp sense of the sacred and anthropocentric. Ubuntu is therefore central to the African way of life and is defined as an inescapable spirit of hospitality and harmony shown to one another (De la Porte 2016; Poovan, Du Toit & Engelbrecht 2006). It is an old African term for ‘humanness’, and it demonstrates the act of sharing, caring and associated values (De la Porte 2016; Nzimakwe 2014). These principles are in keeping with the rationale for healthcare professionals to be caring.

## Problem statement

While healthcare is constantly evolving with technology, there has also been a shift to promote a more patient-centred caring environment. Thus, sonographers need to also adapt to this new environment. But for sonographers to be properly equipped for this patient-centred environment, there is a need to first explore what caring actually means in the context of sonography. Research studies have explored the experiences of caring in other healthcare professionals, but limited attempt has been made to explore a sonographer’s experience of being a caring professional. Hence, this study aimed to explore and describe the sonographers’ experiences of being a caring professional.

## Methodology

### Research design and method

This study employed a qualitative, exploratory, descriptive and contextual research design. The motive of the researchers was to understand, explore and describe the ‘lived experiences’ of the participants (Edmonds & Kennedy 2017). The qualitative descriptive design allowed the participants to describe in words their experiences of being a caring professional. The research design was applied in two phases. In phase 1, sonographers’ experiences of being a caring professional were explored and described through focus group interviews with sonographers working in private practices in Gauteng.

Phase 2 focused on the development of guidelines. However, phase 2 will not be discussed in this journal article.

### Research population and sample

The population included all sonographers registered with the Health Professions Council of South Africa (HPCSA) working in private healthcare settings in Gauteng. Sonographers were invited to participate in this study through purposive snowball sampling technique to ensure information-rich data. The researcher was acquainted with the participants of the first focus group. These participants carried knowledge about sonography and were practising in a private healthcare setting, hence meeting the inclusion criteria. Thereafter, sonographers who also met the inclusion criteria were referred by the initial participants of the study. The researcher made a conscious decision to self-monitor her own actions in order to reduce research bias and ultimately ensure credibility (Holloway & Wheeler 2010:8). The researcher made use of reflexivity by keeping reflective field notes throughout the data collection process. The sample size was dependent on data saturation. A total of 14 participants were included in four focus group interviews.

### Data collection

An information letter was given to each participant to explain the background of, rationale for and purpose of the research study. Informed consent for interviewing the participants and the use of a tape recorder was obtained before conducting the interviews. The focus group interviews were conducted in a neutral environment outside the radiology department. This method is more advantageous because it is socially orientated, and participants are more relaxed than with one-on-one interviews (Marshall & Rossman 2016).

Four focus group interviews were conducted in English, and the groups ranged between three and five participants each. The venues were spacious enough to accommodate the participants but small enough to easily pass the audio recorder around. The research question was as follows: *Tell me about being a caring professional in sonography*. The researcher used interviewing techniques (including confirmation, probing and paraphrasing) to add value to the interviews and to gain more insight into sonographers’ experiences of being caring professionals (Brink 2010; Ramlaul 2010). During this study, bracketing was applied to set aside the researcher’s feelings and opinions, while gaining an in-depth understanding of a sonographer’s experiences of being a caring professional (Tuophy et al. 2013). Extensive field notes were taken on additional information such as body language and group dynamics (Flick 2014).

### Analysis

The transcribed data alongside the documented field notes were analysed using thematic data analysis, which is a method of identifying, analysing and reporting themes (Castleberry & Nolen 2018). The researcher used the analysis process of Holloway and Wheeler (2010) to sort the data into themes and categories. The steps of Holloway and Wheeler are as follows: transcribing interviews and sorting field notes; organising, ordering and sorting the data; listening to and reading or interviewing the material collected repeatedly; coding and categorising; building themes; and describing a phenomenon.

### Trustworthiness

Trustworthiness in qualitative research is described as a methodological adequacy and accuracy (Holloway & Wheeler 2010). Credibility was achieved through peer debriefing, triangulation and reflexivity. Well-organised methods and audit trails were established and used to ensure dependability (Holloway & Wheeler 2010). Transferability was achieved by producing analytic summaries and verbatim quotes, together with a thorough description of the research setting and data population. Confirmability was ensured by a confirmability audit. This audit included audiotape recordings, coding details and field notes to confirm the study’s findings.

## Ethical considerations

The four principles for resolving ethical considerations, that is, respect for autonomy, beneficence, justice and nonmaleficence, were applied throughout the study (Dhai & Mason 2011). This was achieved by reminding the participants that their participation was voluntary and that they were entitled to withdraw at any time without penalty. The audio recordings remained anonymous and were kept in a locked safe. This will be destroyed after 2 years. The information obtained from the focus group interviews was only accessible to the researcher, supervisors and transcribers, who also signed a consent form for confidentiality. In addition, data collection followed only after ethical approval was granted by the Research Ethics Committee (REC-01-34-2018) of the University of Johannesburg.

## Research findings and discussion

Four themes emerged after the data analysis was completed: the effects of a caring relationship between a sonographer and a patient; circumstances limiting a sonographer in being a caring professional; a sonographer’s approach to caring; and the educational readiness of sonographers to be caring professionals.

### Theme 1: The effects of a caring relationship between a sonographer and a patient

It was evident throughout the interviews that there was a unique relationship between the sonographer and the patient. However, despite a sonographer’s willingness to care, there were still numerous effects, both positive and negative, that influenced a sonographer’s caring ability, for example, with regard to positive effects, constructive advantages such as caring relationships that harvest and enhance trust between the sonographer and patient. Further, the negative effects can include the inability of sonographers to practice emotional distancing from their patients and keeping a balance between caring and performing the ultrasound examination. The caring relationship amongst patient and sonographer enhances trust and assurance. The participants’ quotes below express the relationship building between a sonographer and a patient:

‘… There was a patient that came for a follow up breast sonar. She came for a routine mammogram and ultrasound and we picked up there was a mass … when she came back now, I did not even realise that I had an impact on her and then she said every time she thinks of something difficult, she thinks of me. [*Emotional*] I did not even realise that I had helped her that much … I was just basically a friend to her in that situation.’ (FG 2, P1, 33 years)

‘I had a kid and I did the ultrasound and she had to come back for a follow-up and as soon as they came through the passage the mom said “there is that nice aunty you talked about. The same aunty is going to help you” and the kiddie was all full of smiles. She was happy she could get on the bed.’ (FG 4, P2, 28 years)

Stories shared by the participants indicated that patients build trust from their caring. These stories are well supported by the literature. The Cambridge Dictionary (2018) defines trust as ‘to believe that someone is good and honest and will not harm you, or that something is safe and reliable’. Trust is an important concept in the healthcare setting, as there already is an element of risk and uncertainty for the patient (Allison & Chaar 2016). Patients will most likely disclose information with a healthcare professional whom they trust. This will lead to improved interaction, better caring perceptions and lower anxiety levels from the patient (Allison & Chaar 2016; Birkhauer et al. 2017).

Furthermore, the participants experienced negative effects of caring in the form of emotional and psychological strain. These effects are highlighted in the verbatim quotes below:

‘Well, I think if you get too involved with your patient also it gets difficult for a sonographer. Especially if it is sad cases … you can’t take all of that on yourself.’ (FG 2, P1, 33 years)

‘So it is a very fine line and I think it is probably one of my biggest struggles personally in the career to keep that balance between caring and doing what I need to do …’ (FG 3, P1, 30 years)

Studies have shown that the emotional and physical needs of patients, job demands and workload are the main cause of stress and poor emotional well-being among healthcare professionals (Font, Corti & Berger 2015; Janjhua & Chandrakanta 2012:110; Santos et al. 2016). Prolonged stress that results in exhaustion and burnout is frequently detected in various professions, including in the healthcare setting (Font et al. 2015). According to Font et al. (2015), emotional exhaustion, low job performance and decreased personal fulfilment are features of burnout. Burnout is a psychological syndrome that is characterised by a decrease in strength and a disconnection from work involvement. It mostly encompasses exhaustion and cynicism (Santos et al. 2016:415). Strain is defined as the physiological balance between environmental demands and personal coping mechanisms (Ashong et al. 2016; Santos et al. 2016; Strubin 2017). The physiological and social composure of a person therefore determines his or her attitude and reaction during stressful encounters (Janjhua & Chandrakanta 2012).

Sonographers are therefore encouraged to have reflective journals to document important events that might have an emotional influence on them. Employers can also invest in building a tea garden for sonographers that can be used when needed, for it has multiple mental well-being benefits. Additionally, a psychologist can be made available to counsel traumatised or emotionally challenged sonographers.

### Theme 2: Circumstances limiting a sonographer in being a caring professional

Theme two emerged after the participants explained that they encountered situations that limit their caring ability. The participants acknowledged that a sonographer should be caring but they showed feelings of concern regarding the negative influence that factors such as time constraints had on their caring ability. In addition, some participants also explained that they were task driven and focused on answering the clinical question first by providing the required diagnostic information. The participant’s stories are shared below:

‘… because that is the most difficult thing for me in a radiology department. There is no time to actually sympathise …’ (FG 2, P3, 36 years)

‘I suppose naturally sonographers should be caring, because we do work with sick patients … we deal with patients who get diagnosed with cancer … terminal illnesses, but I think we have time constraints.’ (FG 1, P1, 30 years)

Workload is described as an aspect of time, complexity and the amount of work that must be performed in a certain time frame (Ross, Rogers & King 2018). Literature suggests that an increased workload and time pressures not only contribute to a lack of empathy but also create a barrier to self-compassion and compassionate care for patients (Ross et al. 2018). Compassionate care is defined as the ability to display understanding, empathy and emotional resonance (Lown, Muncer & Chadwick 2015). When a healthcare professional’s time is constrained, they make use of ‘rationing’ by deliberately deciding how much care they can provide in a short period of time (Ross et al. 2018). This was corroborated by the participants’ stories that revealed concern and guilt for failure to provide sufficient care for their patients when their time was limited.

Participants also expressed the importance of focusing on the examination that enabled them to answer the clinical question. The participants felt that they were being caring by performing the examination that was required of them. The quotes below support the task-driven qualities of the participants:

‘I think first of all it is important to answer the clinical question, because they were sent to you for a specific reason … So for me, first of all I focus on the act of actually doing the ultrasound, because they were sent to you for a purpose … to actually answer the clinical question …’ (FG 4, P3, 30 years)

‘If you really get too involved with the patient, then you get to a point where you get too emotional to really deliver the service that you should.’ (FG 2, P2, 31 years)

It is evident through the stories of the participants that a sonographer’s priority is to provide the patient with a diagnosis first, thus leaving room for compromised care. Literature articulates that the advances in technology influence a healthcare professional’s caring ability (Reeves & Decker 2012). Radiology departments are notorious for substituting humanistic interaction of patients by rather focusing on diagnostic excellence (Reeves & Decker 2012). When healthcare professionals focus on operating equipment to demonstrate their technical capabilities and efficiency, it is often mistaken for compromising the emotional aspect of caring (Reeves & Decker 2012). Kagan and Melendez-Torres (2015) are of the opinion that healthcare professionals can easily focus on diagnoses, room numbers and examinations while neglecting their caring for the patient. It is recommended to engage in peer discussions so that sonographers can share experiences and recommendations for caring. In addition, sonography practices should also focus on selecting senior sonographers who display, reflect and encourage caring.

### Theme 3: Sonographer’s approach to caring

Theme three focuses on how sonographers approach caring. The participants felt that there were physical and emotional aspects to caring. Sonographers’ understanding of physical caring is shared in their stories below:

‘I think there are different aspects to it. So there would be physical caring. So patients who need to be helped, you need to help them on and off the bed and stuff …’ (FG 1, P2, 37 years)

‘You know, if they need physical help, I will I help them undress. If a lady can’t walk, I will fetch her in reception and I will lead her, help her undress, get her comfortable on the bed and warm the gel.’ (FG 1, P1, 30 years)

‘Just taking your time to lift up the foot flaps on the wheelchair and taking the gowns off and helping them with the drip and sometimes that you know, it requires patience, especially if they are very immobile and that kind of thing, but I think they appreciate that.’ (FG 1, P3, 39 years)

Technology has improved to benefit the patient. However, patients do not always see these advances and would therefore recognise caring in more emotional components (McMaster & DeGiobbi 2016). Literature shows that the physical aspect of caring involves activities such as giving bed baths and providing medical information (Verhovsek, Byington & Deshkulkarni 2009; Zamanzadeh et al. 2010). McMaster and DeGiobbi (2016) emphasise that little gestures such as a smile, a warm blanket and a reassuring touch have an immense impact on patients. Similarly, Albertina Sisulu, a South African nurse and activist, believed in expressing caring through an act of kindness and a smile (Downing & Hastings-Tolsma 2016). These are ways of creating an environment of respect and caring for the human body, as illustrated by the participants in this study. The participants’ interpretation of physical caring echoes what current literature reveals.

Participants also shared their understanding of emotional caring, such as communication, sympathy and patience. The following quotations depict this:

‘I think communication is very important. If you say to a patient this who I am. This is why you are here and this is what this is what we are going to do.’ (FG 1, P5, 38 years)

‘I think the most important thing is you have to be patient … if you lose your temper with the patient, then the whole thing just goes haywire. You are not focusing on your examination and you miss pathology.’ (FG 3, P3, 36 years)

Healthcare workers generally have a very high emotional and stressful responsibility because of the demand for delivering high-quality healthcare services (Berry, Davis & Wilmet 2015; Janjhua & Chandrakanta 2012). Emotional caring encompasses expressive aspects such as providing emotional support to the patient through offers of confidence and hope (Karlou, Papathanassoglou & Patiraki 2015; Verhovsek et al. 2009; Widmark-Petersson, Von Esson & Sjoden 1998; Zamanzadeh et al. 2010). In addition, Milne and Spuur (2009) maintain that comprehensive listening and the awareness of the verbal as well as the non-verbal needs of patients are essential. Empathy, hope and compassion are behaviours that are associated with caring (Karlou et al. 2015; Naidoo, Lawrence & Stein 2018). The understanding of emotional caring that was expressed by the participants of this study is consistent with the literature. A possible recommendation for sonography education is that higher education institutions could adapt and endorse practices of Jean Watson’s theory of transpersonal caring. The core principles of Watson’s human caring science emphasise the act of exercising loving-kindness while fostering a wholeness of one’s mind, body, and spirit (Naidoo et al. 2018; Pajnkihar, Stiglic & Vrbnjak 2017; Watson Caring Science Institute 2019).

### Theme 4: Educational readiness of sonographers to be caring professionals

The scope of a sonographer is a section which most patients frequently struggle to understand. This grey area causes challenges for both the sonographer and the patient. The participants suggested that the scope of practice of a sonographer should be explained to patients. They felt that their inability and authorisation to disclose any results had an undesirable impact on their caring. The participants’ stories are shared below:

‘I think it is important to start at the beginning of exactly what the role of a sonographer is and I find that that is a problem for most patients and for sonographers themselves. You are not the doctor. So it is very difficult to diagnose. You see a miscarriage or you see that there is an absent foetal heart and the patient can see the same thing that you are looking at and you are not allowed to confirm. At the same time, you are not a nurse. So you are not doing all the detailed caring. That takes time, you know? You spend an entire day with a patient. So you have that bond that you have created. We are sort of in the middle of all of that. We don’t have the time to spend with patients and we don’t have the ability to diagnose. So if you understand your role, that I am here to do this job and the patient understands that, then I think you will be able to deliver a service …’ (FG 1, P5, 38 years)

‘It is a position that you are there for in your current job situation. Those are the rules of the company. You do not divulge information to your patient, even if they ask for it. You don’t know how a patient is going to react. If you tell a patient listen, I see there is some cancer in your liver and the patient walks out there and steps in front of a car, it is your responsibility, because it is not done in the correct setting.’ (FG 3, P3, 36 years)

Sonographers’ continuous interaction with patients may result in the potential to share confidential information and disclose results (Denney-Koelsch, Cote-Arsenault & Lemcke-Berno 2015). However, sonographers who participated in this study have not been trained in applied psychology, which is essential for counselling patients after results are disclosed. The lack of communication between sonographers and patients may have an influence on patient safety, because the sonographers’ response to patient diagnosis may vary according to their experience (Berglund et al. 2012; Denney-Koelsch et al. 2015). The Health Professions Act of South Africa (2016) does not clearly stipulate that it is within the scope of practice for sonographers to disclose information accurately.

Participants shared their concerns and desire to have a psychology module in their training that may aid in dealing with complex situations. Some participants also explained that it was difficult for them to erect barriers to avoid becoming emotionally involved with patients. The quotes below support these findings:

‘I think I often felt or been in circumstances when I feel I am actually out of my depth. It is more the emotional support and I have often felt like this patient needs help and I can’t provide it. So I think that there should be some kind of psychology component to the training that helps prepare us for all of those different situations that are so difficult.’ (FG 1, P3, 39 years)

‘… it is very hard to create that line and that barrier between you and your patient …’ (FG 3, P2, 37 years)

Work-related psychological well-being is described as an individual’s positive experience at work (Mclnerney et al. 2018). There is no universal definition of well-being, although it provides necessary information about one’s coping mechanism and the ability to handle stress (Mclnerney et al. 2018). When healthcare professionals in a good psychological and emotional state deliver high-quality medical care, it can improve the health outcome of patients (Janjhua & Chandrakanta 2012). The lack of preparation for outrageous patient situations contributes to emotional challenges and creates an element of apathy (Arieli 2013; Strubin 2017). On the contrary, the participants did not reference any dangers to patient safety or behaviours of apathy as a result of reduced psychological well-being and therefore did not resonate with current literature. However, although the participants had difficulty in coping with certain challenging scenarios, they were not ignorant of the fact that they might need some form of coping mechanism. It is recommended that communication skills should be enhanced to assist in transferring and disclosing information to patients in the correct manner. A communication module would therefore be of benefit. It is also suggested that positive psychology interventions or Applied Psychology as a subject should be implemented from first-year undergraduate studies. In addition, information on enhancing and rectifying the emotional state of sonographers who did not study Applied Psychology during their postgraduate studies should be considered.

## Limitations of the study

This study was contextual in nature, and therefore, the generalisation of the findings is limited. Furthermore, this study only comprised of participants who were employed within the private sector; however, this was appropriate for this study because they were information-rich participants.

## Conclusion

The purpose of the descriptive phenomenological study was to explore and describe sonographers’ experiences of being caring professionals within private practice in Gauteng. The participants in this study explained and shared their understanding of being caring professionals. They shared many stories and personal experiences regarding trusting relationships with patients. However, as a caring professional many challenges were encountered, both physically and emotionally. It is crucial to recognise these challenges and the importance of improving the well-being of the sonographer. This will ensure a healthy and happy sonographer who will ultimately exhibit sufficient caring towards patients and thus enhance patient satisfaction. Possible recommendations were provided that might not only improve caring among sonographers but also enhance the psychological well-being of healthcare professionals; thus, the implementation of these guidelines is recommended. It is the responsibility of sonographers, sonography departments and universities to implement, enhance and sustain caring within healthcare.
